# Supplementary material for: Advancing genetic pathogenicity prediction with three-dimensional proteoform-phenotype analysis
Source: medRxiv. 2026 Oct 9:2026.10.07.26364649. Preprint. [Version 1] doi: 10.64898/2026.10.07.26364649 (PMC13662702; doi:10.64898/2026.10.07.26364649)
Supplement: Supplementary file 1 [file NIHPP2026.10.07.26364649V1-supplement-1.pdf]

medRxiv preprint doi: https://doi.org/10.64898/2026.10.07.26364649; this version posted October 9, 2026. The copyright holder for this preprint (which was not certified by peer review) is the author/funder, who has granted medRxiv a license to display the preprint in perpetuity.
This article is a US Government work. It is not subject to copyright under 17 USC 105 and is also made available for use under a CC0 license.

Supplementary Materials

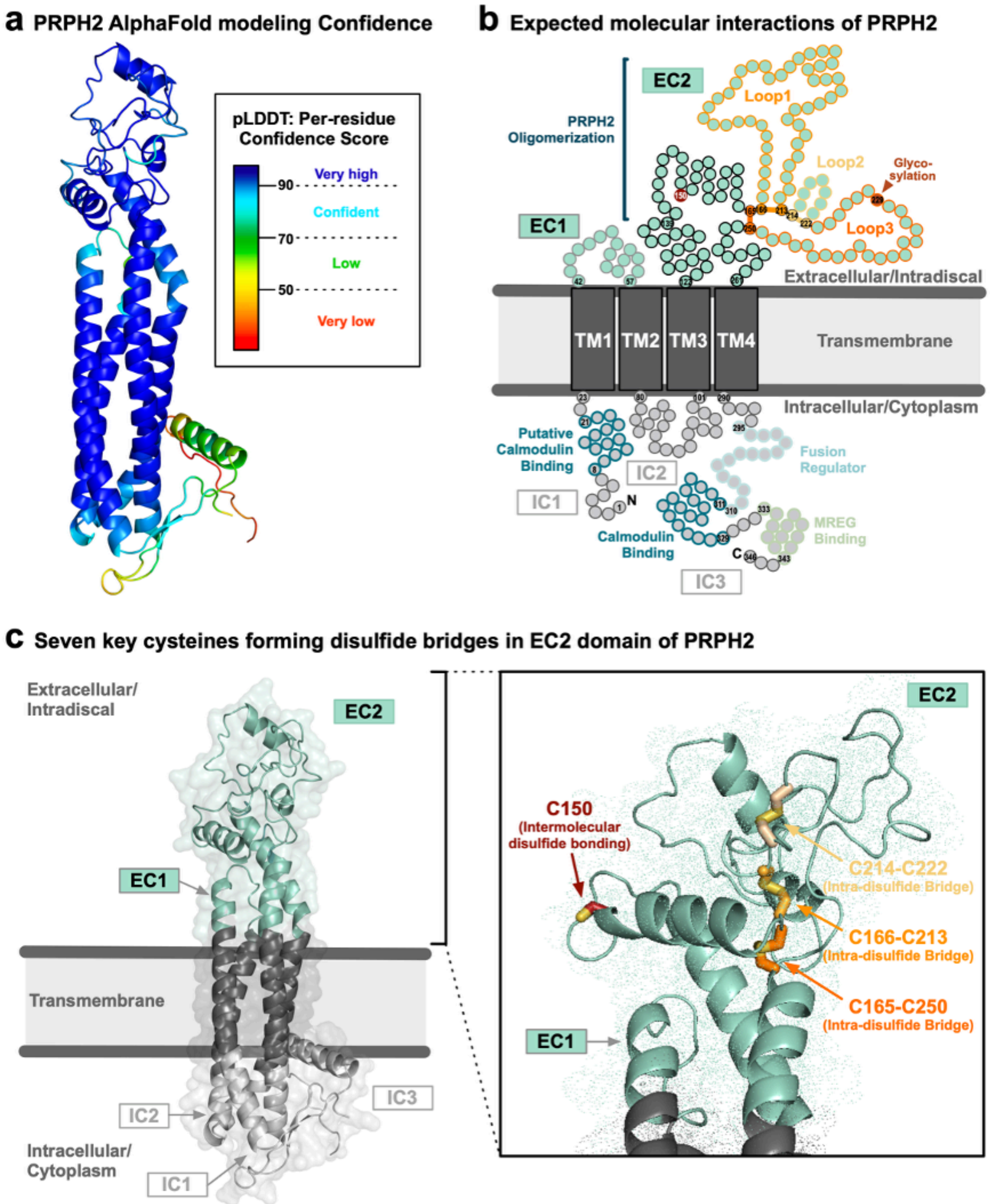

Supplemental Figure 1. Quality assessment of PRPH2 AlphaFold model. a, Modelling confidence of PRPH2 AlphaFold model (AlphaFold ID: AF-P23942-F1) shows that overall PRPH2 AlphaFold model is predicted in very high confidence except the extreme N-termini and C-termini. The predicted structure of residues with per-residue confidence score (pLDDT) greater than 90 are very high in confidence (colored in deep blue). Residues with pLDDT between 70 and 90 are considered to be confidently modelled (colored in cyan). Residues with pLDDT between 50 to 70 are low in confidence. Residues with pLDDT lower than 50 are very low in modeling confidence. b, 2D domain map with anticipated domains and key molecular interactions based on prior studies. The three intramolecular disulfide bridges form three distinctive loops of EC2 domain. c, PRPH2 AlphaFold model properly modelled the three intramolecular disulfide bridges forming EC2 loops. Cys150, intermolecular disulfide bridging cysteine, is also modeled at the solvent exposed area as expected.

medRxiv preprint doi: https://doi.org/10.64898/2026.10.07.26364649; this version posted October 9, 2026. The copyright holder for this preprint (which was not certified by peer review) is the author/funder, who has granted medRxiv a license to display the preprint in perpetuity.
This article is a US Government work. It is not subject to copyright under 17 USC 105 and is also made available for use under a CC0 license.

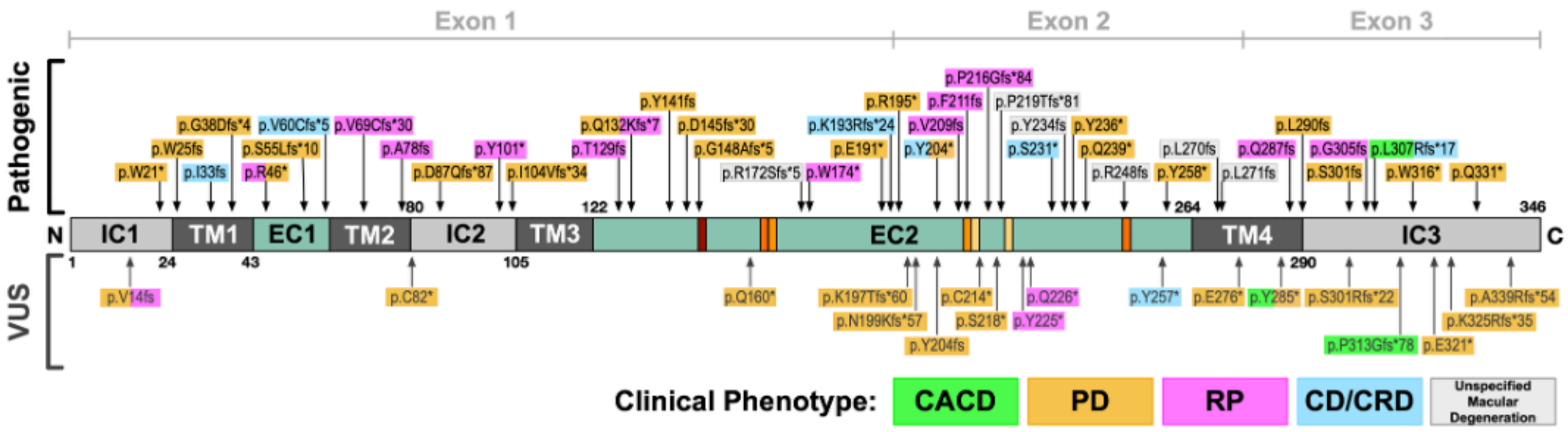

Supplemental Figure 2. Linear mapping of nonsense and frameshift PRPH2 variants. Previously reported PRPH2 nonsense and frameshift variants are mapped to a linear schematic (primary structure map; domain topology map) of the PRPH2 protein. Pathogenic variants and VUS are shown above and under the map, respectively. Variants shown in orange, magenta, green, cyan, and gray boxes were identified in the patients displaying PD, RP, CACD, CD/CRD, and unspecified macular degeneration, respectively.

medRxiv preprint doi: https://doi.org/10.64898/2026.10.07.26364649; this version posted October 9, 2026. The copyright holder for this preprint (which was not certified by peer review) is the author/funder, who has granted medRxiv a license to display the preprint in perpetuity. This article is a US Government work. It is not subject to copyright under 17 USC 105 and is also made available for use under a CC0 license.

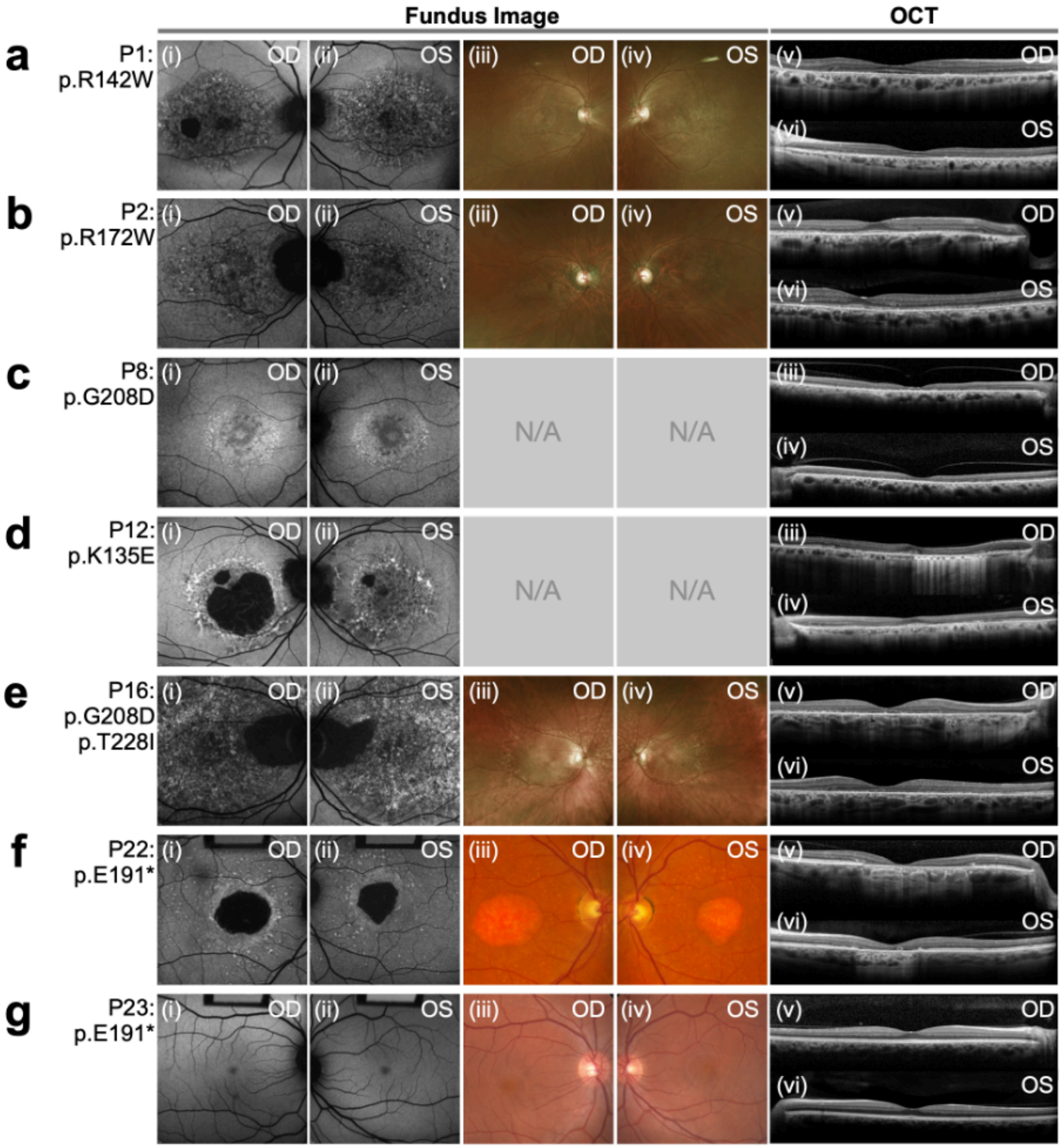

Supplemental Figure 3. Clinical features of patients with central areolar choroidal dystrophy (CACD). a, P1 (p.R142W) displays classic signs of CACD with a speckled fundus autofluorescence (FAF) pattern and granular central macular atrophy with punctate pale yellow lesions OU. b, Clinical imaging of P2 (p.R172W) shows the classic CACD phenotype including speckled pattern of hypofluorescence OU, agranular appearance of the macula along with some peripapillary atrophy, and slight loss of outer retinal structure on OCT. c, FAF of P8 (p.G208D and VUS p.T228I) shows an area of classically speckled AF with peripapillary involvement. Wide-angle fundus photography reveals diffuse yellow deposits in the posterior pole that were absent on FAF. OCT shows increased hyperreflectivity in the outer retinal area. d, FAF for Patient 12 (P12; p.K135E) shows a well-demarcated area of decreased autofluorescence along with a prominent paracentral hyperfluorescent ring OD and a speckled pattern of AF OS. OCT shows bilateral loss of outer retinal layers and disorganization of retinal layers OU along with hyperfluorescent lesions in the retinal pigment epithelium (RPE) OD. e, FAF of P16 (p.G208D) reveals a bullseye pattern of atrophy with refractile crystals and macular atrophy on OCT. f, FAF, fundus photography, and OCT of P22 (p.E191*) shows well-demarcated area of central macular atrophy bilaterally along with a paracentral hyperfluorescent ring OD and hyperfluorescent flecks OU. g, FAF, fundus photography, and OCT for P23 (also with p.E191*) are unremarkable.

medRxiv preprint doi: https://doi.org/10.64898/2026.10.07.26364649; this version posted October 9, 2026. The copyright holder for this preprint (which was not certified by peer review) is the author/funder, who has granted medRxiv a license to display the preprint in perpetuity. This article is a US Government work. It is not subject to copyright under 17 USC 105 and is also made available for use under a CC0 license.

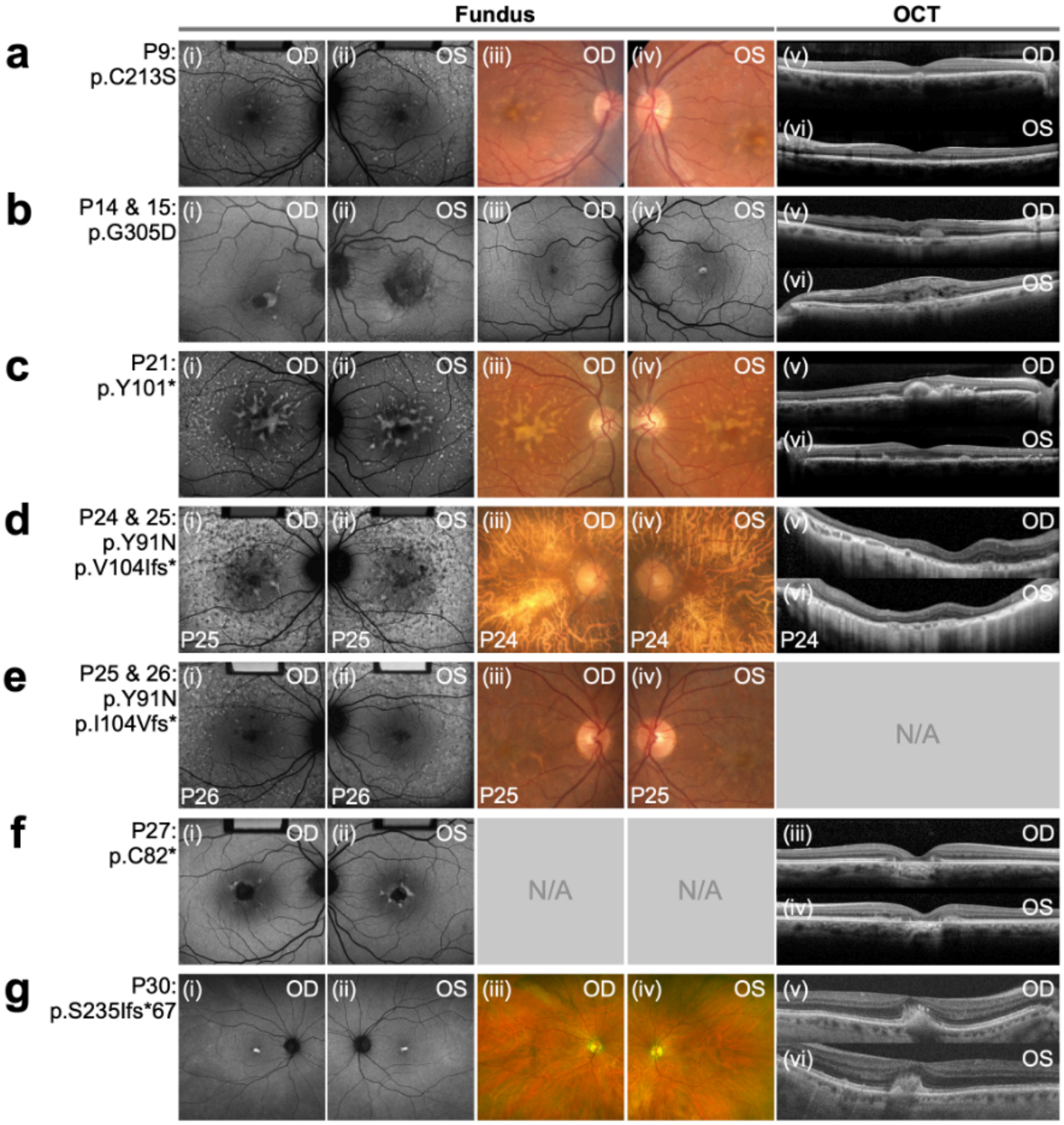

Supplemental Figure 4. Clinical features of patients with pattern dystrophy (PD). a, FAF of P9 (p.C213S) displayed bilateral fleck-like lesions and yellow deposits in the macula were seen in the fundus images. OCT scan of the right eye showed drusen and mostly intact retinal layers. b, FAF OD (i) of P14 (p.G305D) demonstrates a well-demarcated, hyperfluorescent lesion in the central macula. FAF OS (ii) shows diffusely mottled hypofluorescence in the left macula. OCT OD (v) of P14 shows the location of the vitelliform material in the subretinal space, nasal to the right fovea. OCT OS (vi) highlights subretinal fibrosis and disorganization of retinal layers directly surrounding the fovea. FAF OU (iii-iv) of P15 (p.G305D) shows a small, hyperfluorescent vitelliform-like lesion in the left fovea. c, Fundus photography of P21 (p.Y101*) showed extensive pisciform lesions which are all hyperfluorescent on AF. Bilateral OCT through the yellow patches shows large hyperfluorescent subretinal, drusen-like deposits involving the fovea. d and e, P24 (p.Y91N and p.I104V*) shows end-stage retinal atrophy phenocopying Stargardt Disease. Fundus photography for P25 and P26 with the same variants displayed bilateral hyperfluorescent flecks around the macula. f, FAF of P27 (p.C82*) showed butterfly-shaped hyperfluorescent lesions surrounding a small area of central RPE atrophy that is more notable OS than OD. Bilateral OCT confirmed deepening of the fovea. g, FAF of P30 (p.S235Ifs*67) shows hyperfluorescent lesions in the fovea OU that are located in the subretinal space as seen on OCT. There are also small, scattered hypofluorescent spots in the periphery OU.

medRxiv preprint doi: https://doi.org/10.64898/2026.10.07.26364649; this version posted October 9, 2026. The copyright holder for this preprint (which was not certified by peer review) is the author/funder, who has granted medRxiv a license to display the preprint in perpetuity. This article is a US Government work. It is not subject to copyright under 17 USC 105 and is also made available for use under a CC0 license.

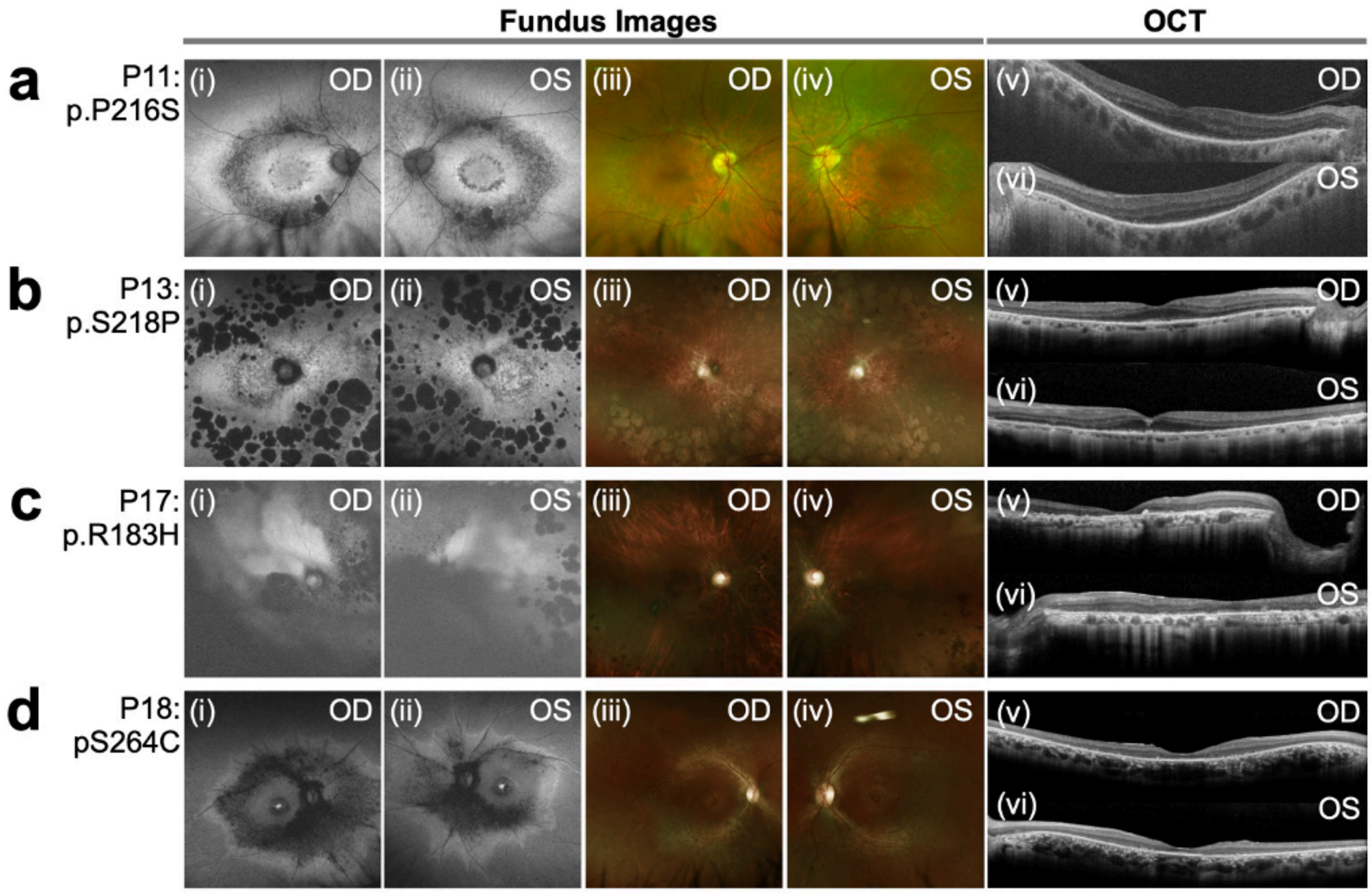

Supplemental Figure 5. Clinical features of patients with retinitis pigmentosa (RP). a, Wide-angled fundus photography, FAF and OCT scan of Patient 11 (P11; p.P216S) showed granular changes along the posterior pole with a distinct ring of atrophy around the macula corresponding to thinning on OCT. b, FAF for P13 (p.S218P) shows granular appearance to the macula with numerous lacunar lesions in the posterior pole and intraretinal pigment migration. c, Wide-angled fundus photography, FAF and OCT scan of P17 (p.R183H) revealed widespread retinal atrophy with superior lacunar degeneration and pigmentary changes in the periphery. d, FAF of P18 (p.S264C) showed granular appearance to the macula with several hypofluorescent lacunar lesions in the posterior pole. OCT scan showed RPE loss.

medRxiv preprint doi: https://doi.org/10.64898/2026.10.07.26364649; this version posted October 9, 2026. The copyright holder for this preprint (which was not certified by peer review) is the author/funder, who has granted medRxiv a license to display the preprint in perpetuity.
This article is a US Government work. It is not subject to copyright under 17 USC 105 and is also made available for use under a CC0 license.

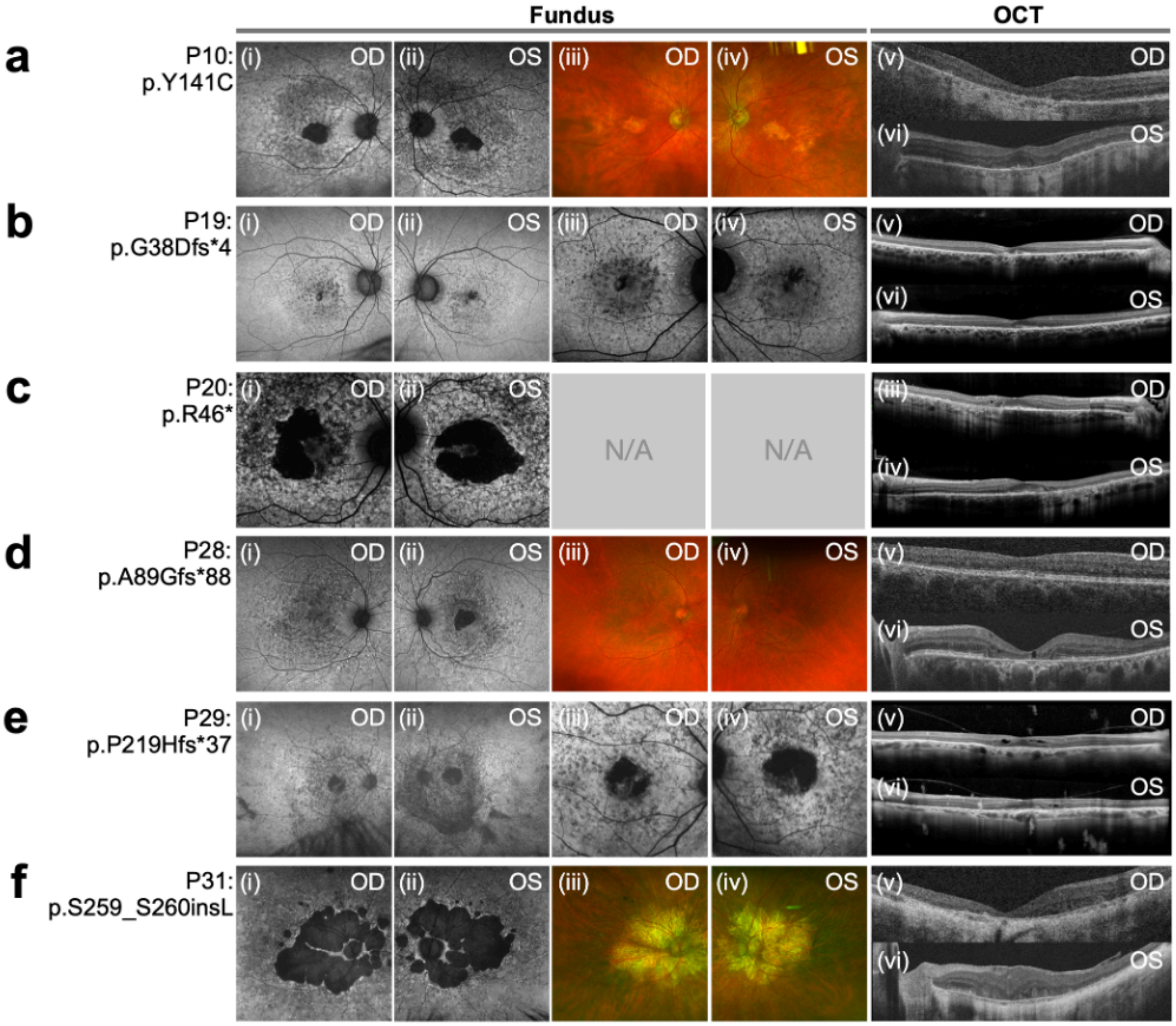

Supplemental Figure 6. Clinical features of patients with cone/cone-rod dystrophy (CD/CRD). a, FAF of P10 (p.Y141C) shows an area of atrophy at the fovea. peripapillary sparing on FAF. OCT scan showed irregular contour, EZ and RPE loss seen in OCT. b, FAF of Patient 19 (P19; p.G38Dfs*4) showed bilateral speckled hypo-autofluorescence that is limited to the central macula. OCT scan revealed some loss of the outer nuclear layer. c, FAF of P20 (p.R46*) showed a well-demarcated area of central macular atrophy with diffuse hypofluorescent specks. OCT scan showed disorganization of retinal layers OU. d, Wide-angle fundus photography and FAF of P28 (p.A89Gfs*88) showed macular pigmentary changes, drusen, granularity at fovea OU, and a pisciform-like lesion in the periphery OU. OCT scan showed distortion of the RPE OS. e, FAF of P29 (p.P219Hfs*37) showed paracentral lesion with jagged border of hypofluorescence around the macula OU. OCT showed intraretinal cysts and partial posterior vitreous detachment (PVD). f, FAF and wide-angle fundus photography of P31 (p.S259_S260insL) revealed severe, diffuse retinal atrophy. Partial peripapillary sparing on FAF. OCT scan showed distortion of the RPE OU.

medRxiv preprint doi: https://doi.org/10.64898/2026.10.07.26364649; this version posted October 9, 2026. The copyright holder for this preprint (which was not certified by peer review) is the author/funder, who has granted medRxiv a license to display the preprint in perpetuity.
This article is a US Government work. It is not subject to copyright under 17 USC 105 and is also made available for use under a CC0 license.

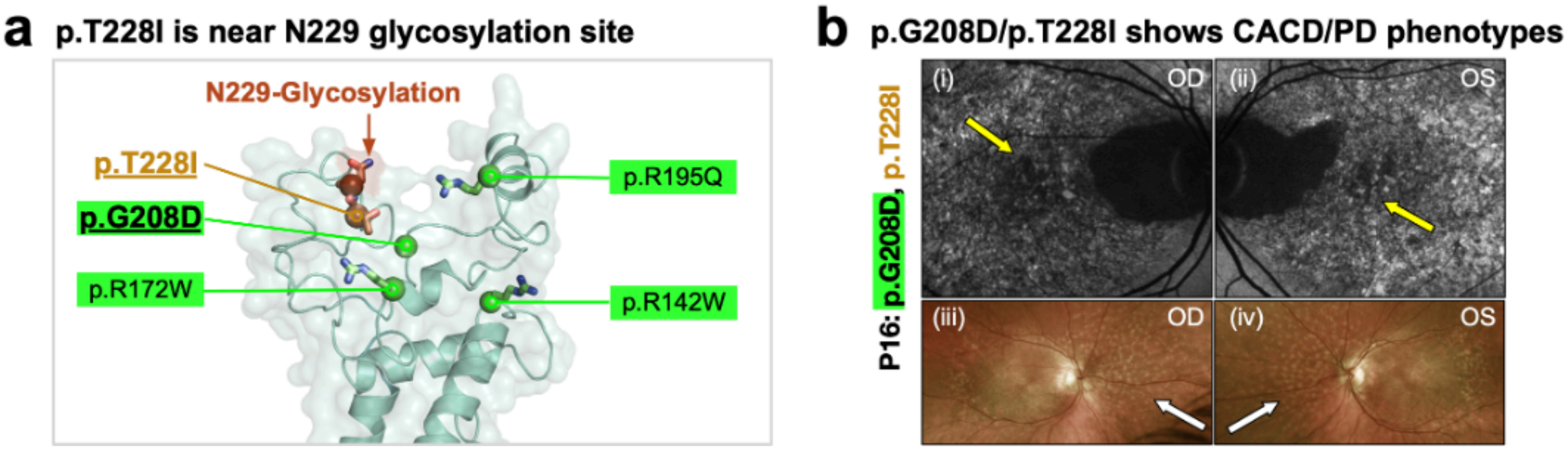

Supplemental Figure 7. Proteotype-phenotype analysis of a patient with two different PRPH2 variants. a, p.T228I, previously reported in PD patients (light brown residue), was right by the Asn229 glycosylation site (brown residue). b, Patient 16 (P16) harboring p.G208D (pathogenic CACD variant) and p.T228I displayed combined phenotypes of CACD and PD. This is illustrated by fundus autofluorescence images (i and ii) showing the speckled pattern of hyper and hypo-autofluorescence typical in CACD, but widefield fundus photographs (iii and iv) showing the yellow fleck-like lesions (white arrows) in the posterior pole typical in STG-PD.

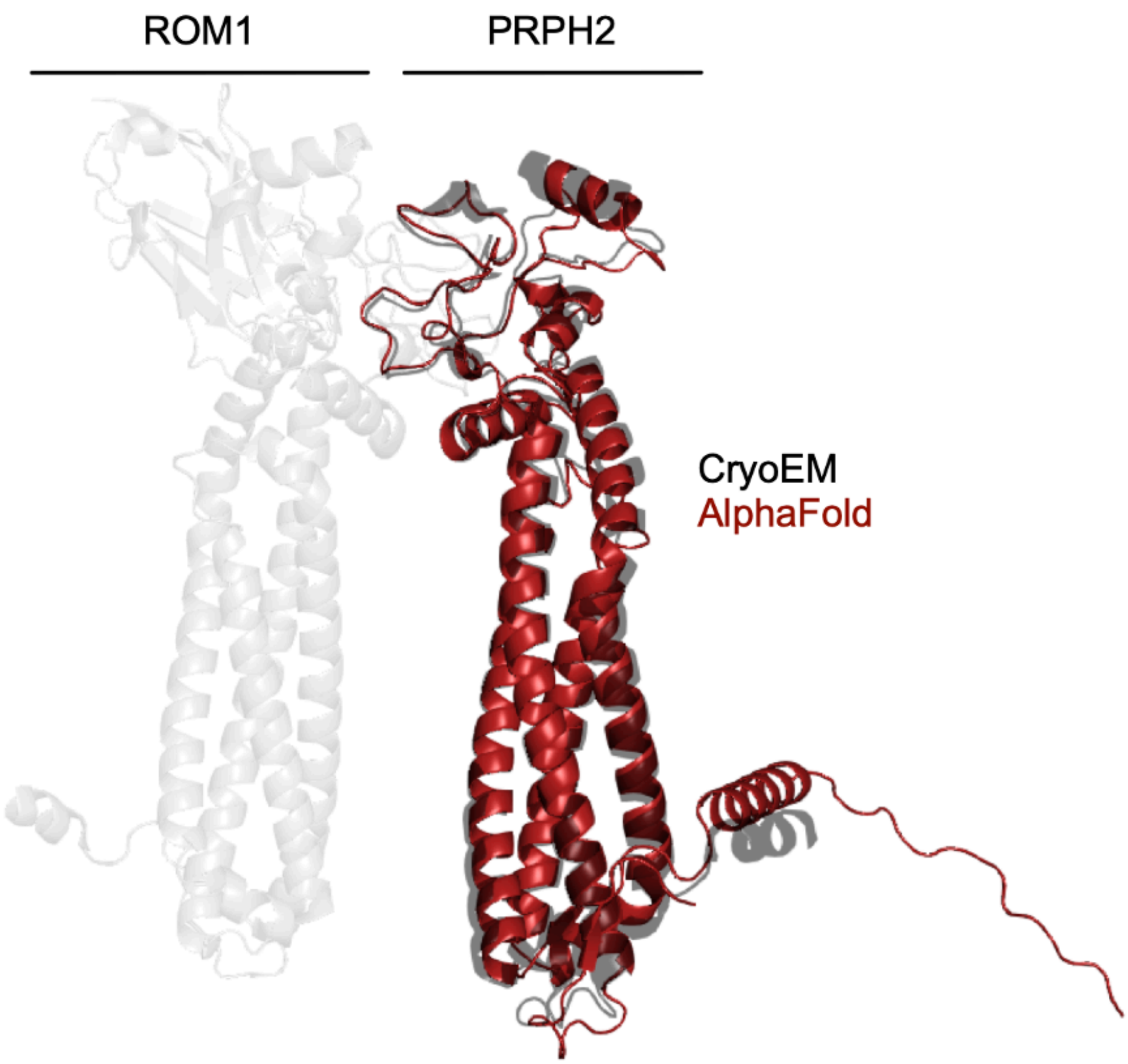

Supplemental Figure 8. Comparison of PRPH2 AlphaFold model to cryogenic electron microscopy structure. Cryogenic electron microscopy structure of PRPH2 in complex with ROM1 validates AlphaFold model. Cα RMSD = 1.397 (2122 to 2122 atoms.)

medRxiv preprint doi: https://doi.org/10.64898/2026.10.07.26364649; this version posted October 9, 2026. The copyright holder for this preprint (which was not certified by peer review) is the author/funder, who has granted medRxiv a license to display the preprint in perpetuity.
This article is a US Government work. It is not subject to copyright under 17 USC 105 and is also made available for use under a CC0 license.

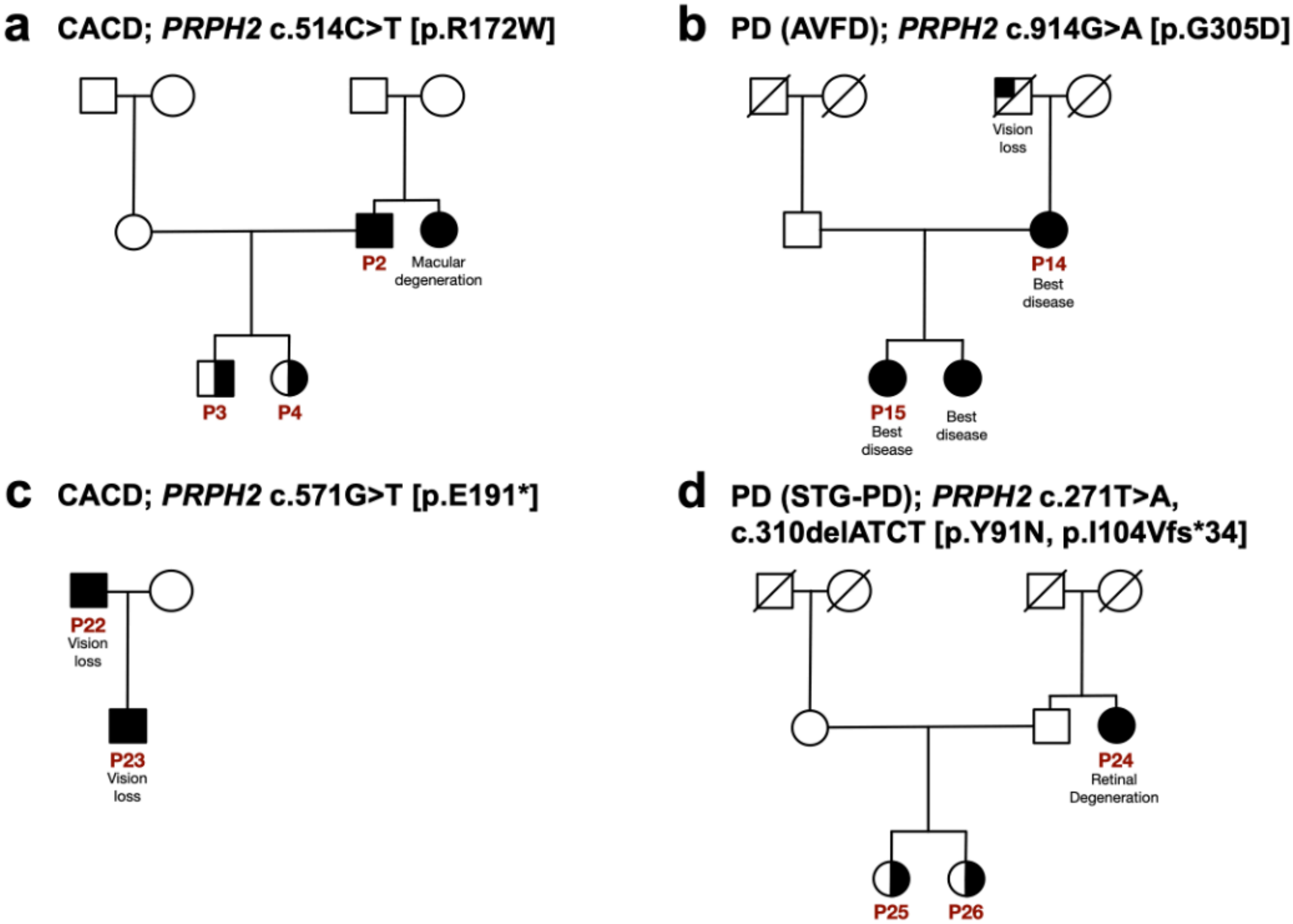

Supplemental Figure 9. Pedigrees of related patients with PRPH2 variants. Multi-generational pedigrees display patterns of inheritance of PRPH2 variants within the same family. Circles represent females, and squares represent males. Symbols with a diagonal line indicate that the person is deceased. Filled symbols indicate that the person is affected with a, PRPH2 c.514C>T [p.R172W], b, PRPH2 c.914G>A [p.G305D], c, PRPH2 c.571G>T [p.E191*], or d, PRPH2 c.271T>A, c.310delATCT [p.Y91N, p.I104Vfs*34].

medRxiv preprint doi: https://doi.org/10.64898/2026.10.07.26364649; this version posted October 9, 2026. The copyright holder for this preprint (which was not certified by peer review) is the author/funder, who has granted medRxiv a license to display the preprint in perpetuity. This article is a US Government work. It is not subject to copyright under 17 USC 105 and is also made available for use under a CC0 license.

Supplemental Table 1. Summary of genotype, proteotype, and phenotype in 31 patients with PRPH2 variants

ID	Age	Sex	BCVA OD	BCVA OS	Genotype	Proteotype	Classification	Phenotype	Structural outcome of mutations	Other identified variants
**Missense Variants**										
1	45 - 49	F	20/80	20/400	c.424C>T	p.R142W	Pathogenic	CACD	Mutation at non-covalent oligomerization interface of EC2	N/A
2*	45 - 49	M	20/20	20/25	c.514C>T	p.R172W	Pathogenic	CACD	Mutation at non-covalent oligomerization interface of EC2	GRM6 c.722-2del, ADGRV1 c.8295G>T (p.L2765F), GNAT2 c.349G>A (p.G117R), MKKS c.1161+4G>A, PRPF4 c.1324G>A (p.V442I), and RP1 c.5522C>T (p.T184I).
3*	15 - 19	M	20/25	20/20	c.514C>T	p.R172W	Pathogenic	CACD	Mutation at non-covalent oligomerization interface of EC2	GRM6 c.722-2del, MKKS c.1161+4G>A, and REEP6 c.298T>C (p.F100L).
4*	10 - 14	F	20/20	20/20	c.514C>T	p.R172W	Pathogenic	CACD	Mutation at non-covalent oligomerization interface of EC2	GRM6 c.722-2del, MKKS c.1161+4G>A, PRPF4 c.1324G>A (p.V442I), and REEP6 c.298T>C (p.F100L).
5	55 - 59	F	20/60	20/60	c.514C>T	p.R172W	Pathogenic	CACD	Mutation at non-covalent oligomerization interface of EC2	N/A
6	25 - 29	M	20/20	20/25	c.514C>T	p.R172W	Pathogenic	CACD	Mutation at non-covalent oligomerization interface of EC2	N/A
7	60 - 64	F	20/100	20/150	c.514C>T	p.R172W	Pathogenic	CACD	Mutation at non-covalent oligomerization interface of EC2	N/A
8	60 - 64	M	20/50	20/60	c.623G>A	p.G208D	Pathogenic	CACD	Mutation at non-covalent oligomerization interface of EC2	N/A
9	35 - 39	F	20/20	20/30	c.638G>C	p.C213S	Pathogenic	PD (STG-PD)	Mutation at C166-C213 intramolecular disulfide bridge	N/A

medRxiv preprint doi: https://doi.org/10.64898/2026.10.07.26364649; this version posted October 9, 2026. The copyright holder for this preprint (which was not certified by peer review) is the author/funder, who has granted medRxiv a license to display the preprint in perpetuity.
This article is a US Government work. It is not subject to copyright under 17 USC 105 and is also made available for use under a CC0 license.

ID	Age	Sex	BCVA OD	BCVA OS	Genotype	Proteotype	Classification	Phenotype	Structural outcome of mutations	Other identified variants
10	75 - 79	M	CF	20/30	c.422A>G	p.Y141C	Pathogenic	CRD	Mutation at covalent oligomerization interface of EC2	N/A
11	55 - 59	F	20/50	20/125	c.646C>T	p.P216S	Pathogenic	RP	Mutation at EC2-loop 2	N/A
12	55 - 59	M	20/25	20/25	c.403A>G	p.K135E	VUS	CACD	Mutation at non-covalent oligomerization interface of EC2	N/A
13	65 - 69	M	20/40	20/40	c.652T>C	p.S218P	VUS	RP	Mutation at EC2-loop 2	N/A
14#	75 - 79	F	20/100	20/125	c.914G>A	p.G305D	VUS	PD (AFVD)	Mutation at fusion regulatory region of IC3	N/A
15#	40 - 44	F	20/20	20/40	c.914G>A	p.G305D	VUS	PD (AFVD)	Mutation at fusion regulatory region of IC3	N/A
16	45 - 49	M	20/200	20/200	c.623G>A, c.683C>T	p.G208D, p.T228I (unknown whether cis or trans)	Pathogenic, VUS	CACD	Mutation at non-covalent oligomerization interface of EC2	N/A
17	50 - 54	F	20/80	20/125	c.548G>A	p.R183H	Novel	RP	Mutation at covalent oligomerization interface of EC2	N/A
18	35 - 39	M	20/80	20/100	c. 791C>G	p.S264C	Novel	RP	Mutation at TM4	ABCA4 (c.2666T>C [p.V889A]).
**Frameshift and Nonsense Variants**										
19	50 - 54	M	20/40	20/30	c.113delG	p.G38Dfs*4	Pathogenic	CRD	Frameshift begins at TM1	N/A
20	35 - 39	M	20/20	20/30	c.136C>T	p.R46*	Pathogenic	CRD	Terminates in EC1	N/A
21	45 - 49	M	20/40	20/25	c.303C>G	p.Y101*	Pathogenic	PD (STG-PD)	Terminates in TM3	N/A
22**	60 - 64	M	20/400	20/80	c.571G>T	p.E191*	Pathogenic	CACD	Terminates in EC2	N/A
23**	35 - 39	M	20/20	20/20	c.571G>T	p.E191*	Pathogenic	Normal	Terminates in EC2	N/A
24##	75 - 79	F	NA	NA	c.271T>A, c.310delATCT	p.Y91N, p.I104Vfs*34 (likely in cis)	VUS, Pathogenic	PD (STG-PD)	Frameshift begins at TM3	N/A
25##	45 - 49	F	20/20	20/40	c.271T>A, c.310delATCT	p.Y91N, p.I104Vfs*34 (likely in cis)	VUS, Pathogenic	PD (STG-PD)	Frameshift begins at TM3	N/A
26##	30 - 34	F	20/20	20/20	c.271T>A, c.310delATCT	p.Y91N, p.I104Vfs*34 (likely in cis)	VUS, Pathogenic	PD (STG-PD)	Frameshift begins at TM3	N/A
27	60 - 64	F	20/40	20/70	c.246C>A	p.C82*	VUS	PD (BPD)	Terminates in IC2	N/A

medRxiv preprint doi: https://doi.org/10.64898/2026.10.07.26364649; this version posted October 9, 2026. The copyright holder for this preprint (which was not certified by peer review) is the author/funder, who has granted medRxiv a license to display the preprint in perpetuity.
This article is a US Government work. It is not subject to copyright under 17 USC 105 and is also made available for use under a CC0 license.

ID	Age	Sex	BCVA OD	BCVA OS	Genotype	Proteotype	Classification	Phenotype	Structural outcome of mutations	Other identified variants
28	70 - 74	M	20/40	20/40	c.265dup	p.A89Gfs*88	Novel	CRD	Frameshift begins at IC2	N/A
29	60 - 64	F	20/50	20/50	c.665delC	p.P219Hfs*37	Novel	CD	Frameshift begins at EC2	N/A
30	60 - 64	F	20/40	20/25	c.700_703dup	p.S235Ifs*67	Novel	PD (AFVD)	Frameshift begins at EC2	N/A
**Insertion Variant**										
31	60 - 64	M	20/250	20/40	c.776_777insTCT	p.S259_S260insL	Novel	CRD	In-frame insertion at the end of the EC2 domain	N/A

Patients with the same symbol (*, **, #, ##) after the ID are part of the same family. Abbreviations: AFVD (adult-onset foveomacular vitelliform dystrophy), BCVA (best corrected visual acuity), BPD (butterfly pattern dystrophy), CACD (Central Areolar Choroidal Dystrophy), CF (count fingers), OD (right eye), OS (left eye), PD (pattern dystrophy), STG-PD (Stargardt's-like pattern dystrophy), NA (not available).
